# Responsiveness and minimal important change of the Family Reported Outcome Measure (FROM-16)

**DOI:** 10.1186/s41687-024-00703-1

**Published:** 2024-03-26

**Authors:** R. Shah, A.Y. Finlay, M.S. Salek, H. Allen, S.J. Nixon, M. Nixon, K. Otwombe, F.M. Ali, J.R. Ingram

**Affiliations:** 1 Division of Infection and Immunity, School of Medicine, Cardiff University, Cardiff, UK; 2 School of Life and Medical Sciences, University of Hertfordshire, Hatfield, UK; 3 Shine Charity, Cardiff, UK; 4 Multiple Sclerosis Society, Cardiff, UK; 5 Statistics and Data Management Centre, Perinatal HIV Research Unit, Chris Hani Baragwanath Academic Hospital, University of the Witwatersrand, Johannesburg, South Africa

**Keywords:** Sensitivity to change, Responsiveness, Longitudinal validity, FROM-16, Change over time, MCID, MIC

## Abstract

**Background:**

The FROM-16 is a generic family quality of life (QoL) instrument that measures the QoL impact of patients’ disease on their family members/partners. The study aimed to assess the responsiveness of FROM-16 to change and determine Minimal Important Change (MIC).

**Methods:**

Responsiveness and MIC for FROM-16 were assessed prospectively with patients and their family members recruited from outpatient departments of the University Hospital Wales and University Hospital Llandough, Cardiff, United Kingdom. Patients completed the EQ-5D-3L and a global severity question (GSQ) online at baseline and at 3-month follow-up. Family members completed FROM-16 at baseline and a Global Rating of Change (GRC) in addition to FROM-16 at follow-up. Responsiveness was assessed using the distribution-based (effect size-ES, standardized response mean -SRM) and anchor-based (area under the receiver operating characteristics curve ROC-AUC) approaches and by testing hypotheses on expected correlation strength between FROM-16 change score and patient assessment tools (GSQ and EQ-5D). Cohen’s criteria were used for assessing ES. The AUC ≥ 0.7 was considered a good measure of responsiveness. MIC was calculated using anchor-based (ROC analysis and adjusted predictive modelling) and distribution methods based on standard deviation (SD) and standard error of the measurement (SEM).

**Results:**

Eighty-three patients with 15 different health conditions and their relatives completed baseline and follow-up questionnaires and were included in the responsiveness analysis. The mean FROM-16 change over 3 months = 1.43 (SD = 4.98). The mean patient EQ-5D change over 3 months = −0.059 (SD = 0.14). The responsiveness analysis showed that the FROM-16 was responsive to change (ES = 0.2, SRM = 0.3; *p* < 0.01). The ES and SRM of FROM-16 change score ranged from small (ES = 0.2; SRM = 0.3) for the distribution-based method to large (ES = 0.8, SRM = 0.85) for anchor-based methods. The AUC value was above 0.7, indicating good responsiveness. There was a significant positive correlation between the FROM-16 change scores and the patient’s disease severity change scores (*p* < 0.001). The MIC analysis was based on data from 100 family members of 100 patients. The MIC value of 4 was suggested for FROM-16.

**Conclusions:**

The results of this study confirm the longitudinal validity of FROM-16 which refers to the degree to which an instrument is able to measure change in the construct to be measured. The results yield a MIC value of 4 for FROM-16. These psychometric attributes of the FROM-16 instrument are useful in both clinical research as well as clinical practice.

**Supplementary Information:**

The online version contains supplementary material available at 10.1186/s41687-024-00703-1.

## Introduction

It is important to be able to validly measure the impact that having a health condition has on the quality of life (QoL) of family members or partner. This is because, if the impact is recognised clinically, tailored and targeted support may subsequently also benefit the patient. Measurement tools could allow researchers to assess whether interventions might improve the QoL of family members. The wider burden of disease also needs to be measured to fully assess the value of new therapies. The Family Reported Outcome Measure (FROM-16) is a generic family QoL instrument designed to measure this impact on family members of a patient, across all disease areas and patient ages.

### Family quality of life

Caring for a family member/partner with a health condition, particularly a chronic one, disrupts normal family life and can trigger feelings of anxiety, depression, anger, fear and helplessness, impacting family QoL [1–4]. Having a family member with a health condition can change the family dynamics, which then impacts the individual family members. Although family QoL is an integrated, unifying family concept, each individual within a family may be affected in a specific way, which may vary from person to person. FROM-16 can assess these individual person experiences.

FROM-16 can be used in routine clinical practice to support family members of patients across all disease specialities. Such information could also be useful for multidisciplinary team meetings when considering individual patients. Additionally, FROM-16 can be used in Value-Based Healthcare (VBHC), a new paradigm for the allocation of healthcare resources, increasingly being embraced across the world [5]. One of the important components included in VBHC is societal value, a key element of which is to measure the impact of a condition (and the gains from treating or controlling the condition) on a person’s family, and FROM-16 is an appropriate tool for this measurement [6, 7]. This means that FROM-16 could be used alongside patient-reported outcome measures (PROMs) to enhance the accuracy of data underpinning VBHC by providing a wider information base for resource allocation. Key psychometric properties of FROM-16 have been demonstrated, such as internal consistency, test-retest validity and reliability [8]. Mapping of FROM-16 scores to EQ-5D-3L utility values is now possible for economic appraisal purposes [9]. Crucially, score-meaning band descriptors have been described [10], potentially transforming FROM-16 into a useful clinical tool. However, it is also important to demonstrate that the measure responds appropriately to QoL change, and also to know the threshold of a minimal change in the family member’s QoL that family members consider important i.e. the minimal important change (MIC), after considering different thresholds. Once these have been established, FROM-16 could be used widely for research and to inform clinical decision taking. This study, therefore, aimed to measure the responsiveness of FROM-16 to change over time and to estimate the MIC.

## Methods

Patients and their family members/partners were recruited from the outpatient clinics of dermatology, diabetology, rheumatology, haematology and gastroenterology at the University Hospital of Wales and the University Hospital Llandough, Cardiff, United Kingdom. This heterogeneity of the patient set in the study is important when testing a generic measure. The study was conducted between August 2022 to April 2023. The data for FROM-16 responsiveness and for estimation of the MIC value was collected at the same time. However, the FROM-16 responsiveness study included patients and family members (aged ≥ 18 years) while the MIC study (family member only study) included the same family members from the responsiveness study and additionally family members of paediatric patients.

All patients and family members gave their electronic informed consent. Only one family member per patient contributed to the study and the family member was chosen by the patient.

We used non-probability purposive sampling where patients were recruited following set inclusion and exclusion criteria. Only those patients who were starting on a new therapy/medication, changing treatment following therapy failure, or patients whose existing treatment was adjusted, and their family members (aged ≥ 18 years) were included in the study. This was to ensure the detection of change between baseline and follow-up as it would have been difficult to see change in family members of stable patients. Family members were excluded if they were aged under 18 years or if they had significant morbidity, because of the possibility that this might confound the findings.

The study was approved by the Health Research Authority (HRA) and Health and Care Research Wales (HCRW) 20/EE/0242.

When measuring change over time, it is important to consider whether the change in a person’s score after an intervention is valid. This implies that the tool should measure the change in the construct under consideration, but it should also measure the correct amount of change, i.e. it should not underestimate or overestimate the actual change that has taken place. This is known as responsiveness or longitudinal validity. Responsiveness should include a longitudinal study design with at least two assessments with time points chosen in such a way that it can be expected that at least a portion of the study population will change regarding the impact of the construct [11]. This study followed COnsensus-based Standards for the selection of health Measurement Instruments (COSMIN) guidelines [11, 12] and the recommendations of Terwee et al [13] for conducting and reporting high-quality responsiveness and MIC studies.

### Study design

This was a longitudinal study. Patients and their family members recruited from the five outpatient clinics were directed to complete an online study pack (i.e. demographics and questionnaires) at baseline and at three months follow-up. At baseline, patients completed basic details (age, gender, ethnicity, occupation, disease diagnosis, whether or not started a new treatment or adjusted medication, and start date), the EQ-5D-3L questionnaire and the global severity question (GSQ) to provide self-assessment of disease severity. The family members completed some basic demographic details (age, gender, ethnicity, occupation and their relationship to patient), and the FROM-16 questionnaire.

At three months follow-up, the patient again completed the EQ-5D-3L and the GSQ . The family member/partner completed the FROM-16 and Global Rating of Change (GRC) question, recording the overall change in their QoL since baseline. The time period of three months was chosen as clinicians expected to see change in QoL of patients during this period following new treatment/therapy. Accordingly, change was expected in at least some of the family members in the construct of interest. However, three months is a short recall period compared to the recall period used in other family member and/or informal carer responsiveness studies [14, 15].

After three months, participants were emailed a link to the follow-up questionnaire and reminded through text message to complete the follow-up questionnaire. To maximise a timely response rate, either a follow-up text message, a phone call or a reminder email was used.

### Family member/partner assessment techniques

#### FROM-16

The FROM-16 is a generic family QoL questionnaire which measures the impact of any disease, across all medical specialities, on the QoL of adult family members or partners of patients of any age [8]. FROM-16 was created following interviews with 133 family members of patients across 26 medical specialities, exploring in depth the impact of a relative’s health condition on family members. The FROM-16 comprises 16 items, each with three response options: ‘Not at All’ (score = 0), ‘A Little’ (=1) and ‘A Lot’ (=2). The 16 items are divided into two categories (domains): Emotional (comprising six items, maximum score of 12) and Personal and Social Life (comprising ten items, maximum score of 20). The key themes include emotional impact (feeling of being worried, sad, frustrated, angry and difficulty in sharing thoughts and caring) and personal and social impact (impact on time for self, travel, eating habits, family activities, sex life, holidays, work and study, family relationships, family expenses, and sleep) [8]. Although FROM-16 has two distinct domains, FROM-16 scores are calculated as a total summary score. Therefore, responsiveness was tested using the total FROM-16 score. The lowest possible score of FROM-16 is 0, and the highest 32. The higher the total score, the greater is the negative effect on the family member’s QoL.

FROM-16 has demonstrated high internal consistency (n = 120, Cronbach’s α = 0.91) and high reproducibility (n = 51, ICC = 0.93), with a mean completion time of two minutes. Construct validity was proven through the correlation between FROM-16 and WHOQOL-BREF total scores (n = 119, *r* = −0.55, *p* < 0.001), and the correlation between FROM-16 and the patient’s overall health score (n = 120, *r* = −0.51, *p* < 0.001) [8].

#### Global rating of change question (GRCQ)

The GRCQ used as an anchor, allows family members to give a self-assessment of the change since baseline assessment in their overall QoL, whether it has improved, remained the same or deteriorated [16]. The GRCQ was generated based on previous research [17, 18]. The GRCQ posed to family members was:

*Thinking about the effect of your family member/partner’s condition on you, how much has your quality of life changed since you first took part in this study?*

**Table:** 

Improved	Same	Deteriorated
1. A tiny bit better	0. About the same	−1. A tiny bit worse
2. A little bit better		−2. A little bit worse
3. Somewhat better		−3. Somewhat worse
4. Moderately better		−4. Moderately worse
5. Quite a bit better		−5. Quite a bit worse
6. A great deal better		−6. A great deal worse
7. A very great deal better		−7. A very great deal worse

The GRCQ has a 15-point scoring system with responses ranging from “a very great deal better” (+7) to “no change” (0) to “a very great deal worse” (−7). Some studies have used a 7-point rating scale for GRCQ [19–21]. However, this study used a 15-point scale as this allows a respondent to record even a very small relative change (i.e. able to discriminate between different levels of improvement or deterioration) [18, 22, 23], resulting in greater sensitivity to change. However, using more response options and having unendorsed levels is very often problematic for anchor-based analyses.

Respondents initially had to choose online from three options, “Improved ”, “The same” or “Deteriorated”. If they chose “Improved” they were then given a further seven options: from “1”a tiny bit better to “7” a very great deal better. If they chose “Deteriorated” they were given a further seven options from “−1” a tiny bit worse to “−7” a very great deal worse. The purpose of this two-step response was to simplify this complicated question to improve the respondents’ understanding. This simplified presentation of anchor question did not affect how anchor data was analysed. The anchor data was analysed as planned following the recommendation given in the recent literature [24] for estimating MIC value using anchor methods. For analysis, the anchor ratings were dichotomised into improved/not improved for ‘MIC improvement’, and deteriorated/not deteriorated for ‘MIC deterioration’.

For MIC improvement, the data relating to ‘improved’ included all positive anchor responses (+1 to +7), coded as “1”, whereas for ‘not improved’ the data included all negative anchor responses (−7 to −1) and ‘About the same’ coded as “0”.

For MIC deterioration, the data relating to ‘deteriorated’ included all negative anchor responses (−7 to −1), coded as “1”, whereas for ‘not deteriorated’ the data included all positive anchor responses (+1 to +7) and ‘About the same’ coded as “0”.

### Patient assessment techniques

#### EQ-5D-3L

The Euroqol five dimension (EQ-5D) is a generic HRQoL questionnaire that measures preferences associated with a particular health state. The EQ-5D-3L consists of five dimensions (mobility, self-care, usual activities, pain, and anxiety), each with three levels (no problem = 1, some problems = 2, and extreme problems = 3). For this study, the index was calculated using the set of specific values (Tariffs) of the EQ-5D-3L UK version [25]. In this tariff, the utility values attached to different EQ-5D health states range from –0.594 to 1, where 1 is defined as perfect health, 0 represents death, and negative values denote health states worse than death.

#### Global severity (GS) scale

The GS scale was used as an anchor to allow patients to give a self-assessment of their disease severity at baseline and at the three-month follow-up. The question asked to the patient was: “*Thinking about your health, on a scale of 0 to 10 how severe do you consider your disease is today?” The patients answered the question on a scale of 0 to 10, with ten being the most severe and zero being the least severe.* This anchor approach was used to test if the QoL of family members/partners changed with changes in patient disease severity.

### Statistical analysis

#### Responsiveness

The normality was assessed by observing histograms, Q–Q plots and statistical method of Skewness and Kurtosis [26, 27]. Parametric (paired *t*-test) and non-parametric Wilcoxon tests were used as appropriate (depending on fulfilment of normality criteria) to assess whether the FROM-16 could detect changes that occurred from baseline to follow-up.

The responsiveness was examined using a construct approach, making informed a priori hypotheses about the direction and magnitude of effect sizes and correlations between the change in FROM-16 scores and the single-item family GRCQ and patient GSQ scores [12, 28, 29]

A distribution-based approach was used to understand the responsiveness of the FROM-16 to change by identifying the magnitude of difference in the FROM-16 score between the baseline and follow-up. The magnitude of the change in the FROM-16 scores was estimated using the Effect size (ES) and Standardized response mean (SRM).

The ES was calculated as a ratio of the raw FROM-16 score difference from the first to the second assessment to the standard deviation at the first assessment. The calculation of change scores used the methodology of Middel and van Sonderen [30]. Effect size index estimates the magnitude of change over time in before-after study designs. As higher scores of FROM-16 indicate a greater negative impact on family members, it is appropriate to use the formula:

$$ES = \,\frac{{n1 - n2}}{{sd1}}$$

where n1 is the baseline FROM-16 score (pre-intervention), n2 is the follow-up FROM-16 score (post intervention) and sd1 refers to standard deviation of baseline scores.

An ES of 0.2 is considered small, 0.5 medium and 0.8 large [31]. The SRM was calculated as the ratio of the raw FROM-16 score difference from the first to the second assessment to the standard deviation of that difference.

Another method for assessing responsiveness involved calculating the area under the receiver operating characteristic (ROC) curve (AUC), which is a measure of the instrument’s ability to discriminate between two groups according to external criteria (in this case, GRC). This method involved dichotomising GRC scores into “improved” against “no improvement” (‘worsening’ and ‘same’ groups) and vice versa and conducting ROC curve analysis. The ‘same’ and the ‘worsened’ groups are incorporated as “no improvement” as family members in both these groups did not observe any improvement in their QoL. The threshold between improved and not improved family members thus uses the entire sample, leading to more reliable estimates. An AUC ≥ 0.7 is considered a good measure of responsiveness.

A change in QoL of family members was hypothesised in relation to change in external HRQoL measures. This hypothesis was tested by assessing the strength of the correlation between family member measures (FROM-16 change score and GRCQ change score) and family member and patient measures (FROM-16 and GSQ change scores), using Pearson’s correlation analyses. Using Cohen’s criteria, absolute values of a correlation between 0.1 and 0.3 are viewed as being “small”, with values between 0.3 and 0.5 considered “moderate” and values above 0.5 as being “large” [31]. A moderate to high correlation was expected between related and similar constructs (FROM-16 and GRCQ), demonstrating convergent validity. A low to moderate correlation was expected between related but dissimilar constructs (FROM-16 and patient measures), demonstrating discriminant validity. This is consistent with Campbell & Fiske [32] who contend that two types of evidence are crucial in the process of validation of a measure as a construct indicator. Responsiveness of FROM-16 to change over time was demonstrated by testing the hypotheses in Table 1:

**Table 1:** Hypotheses for testing responsiveness

1.	An improvement/deterioration in family members’ QoL measured by FROM-16 in relation to a significant improvement/deterioration in patient HRQoL measured by EQ-5D (including when a patient’s health improves or worsens). A higher score of FROM-16 means deterioration while a higher score on EQ-5D-3L means improvement and vice versa)
2.	Moderate to high positive correlation between FROM-16 change scores and the GRC scale measuring a similar construct
3.	Low to moderate positive correlation between the FROM-16 change score and the patient’s disease severity change score measuring dissimilar construct
4.	Low to moderate negative correlation between the FROM-16 change score and the patient’s EQ-5D change score measuring related but dissimilar construct
5.	Family members/informal carers indicating improvement on the associated GRC scale should have a positive mean change score
6.	Family members/informal carers indicating worsening on the associated GRC scale should have a negative mean change score
7.	The mean change score of family members/partners indicating improvement should be higher than the mean change score of unchanged family members/partners, which in turn should be higher than the mean change score of worsened family members/partners
8.	FROM-16 change score (Improvement)^a^, AUC ≥ 7
9.	FROM-16 change score (deterioration)^b^, AUC ≥ 7

^a^based on the anchor perceived improvement; ^b^based on the anchor perceived deterioration

The responsiveness was considered sufficient if ≥75% of the hypotheses were confirmed [29]

#### Minimal important change

The MIC was estimated using anchor-based methods (ROC analysis, adjusted predictive modelling) and distribution-based methods (0.33 SD, 1 SEM and 1.96 SEM). While an anchor-based approach was used as the primary method for calculation of MIC, the distribution-based method was used to provide supportive evidence to choose an MIC value above measurement error. The ROC analysis involved dichotomising anchor responses into “improved” against “no change” (‘worsening’ and ‘same’ groups) and vice versa and conducting ROC curve analysis. The dichotomies were determined following recent literature on MIC estimates of PROMIS measures [13]. The cutoff score where sensitivity and specificity were maximised (known as the Youden index, J) represented the MIC value. The MIC value based on the Youden index ensures that misclassification ([1-sensitivity] + [1-specificity]) is the smallest [33]. Precision was indicated by the AUC value.

Predictive modelling was carried out using logistic regression formula using the same grouping used for the ROC analysis [34].

$$MI{C_{predict}} = \left( {\log \left( {Odd{s_{pre}}} \right) - C} \right)/B$$

Where MIC_predict_ = predictive minimal important change (the MIC value calculated using predictive modelling), C is a constant, also known as the intercept, and B is the regression coefficient for improvement/deterioration.

The adjusted MIC_pred_ was calculated using the formula below if the number of responses of change versus no change was more or less than 50%, following the formula of Terwee et al. [13].

$$MI{C_{predict(adjusted)}} = MI{C_{predict}} - \left( {0.090 + 0.103*Cor} \right)*S{D_{change}}*\log \left( {odd{s_{pre}}} \right)$$

Where MIC_predict_ _(adjusted)_ = adjusted predictive minimal important change; Cor = correlation between the PROMIS (FROM-16) change score and the anchor; SD_change_ = standard deviation of the PROMIS (FROM-16) change score; log-odds(pred)imp = log-odds of improvement = natural logarithm of [proportion improved/(1 − proportion improved)] [34].

The confidence interval was calculated substituting regression values (values for Constant “C” coefficient “B”, standard errors for constant and B, correlation coefficient between constant and B) into Terluin et al’s [35] Excel formula sheet (supplementary material). All confidence intervals (CI) were determined at 95% (Figs. S1–S4).

Floor and ceiling effects were considered to be present if the lowest or highest possible score was achieved by more than 15% of the family members/partners [36]. The analysis was performed using the software IBM SPSS version 27.

## Results

### Demographic characteristics of the study participants

The participants for the responsiveness study included patients and their family members/partners while the participants for MIC included only family members/partners (Table 2)

**Table 2:** Socio-demographic and quality of life score of patients and their family members who completed baseline and follow-up questionnaires for responsiveness and MIC studies

Characteristic		Number (%) or Mean (SD)	**Number (%) or Mean (**SD)
		Responsiveness (n = 83)	MIC (n = 100)
**Patient**			
Age	Mean age	50.99 (18.71)	44.12 (22.94)
	Range	18–89	1–89
Gender	Male	40 (48.2)	48 (48)
	Female	43 (51.8)	52 (52)
Ethnicity	White	74 (89.2)	81 (81)
	Asian/Asian British	6 (7.2)	15 (15)
	Black/African/Caribbean/Black British	2 (2.4)	2 (2)
	Prefer not to say	1 (1.2)	2 (2)
Occupation	In paid work	44 (53.0)	44 (44)
	Unemployed	5 (6.0)	5 (5)
	Homemaker	6 (7.2)	6 (6)
	Retired	25 (30.1)	25 (25)
	Rather not say	3 (3.6)	3 (3)
	NA		17^a^ (17)
Heath condition	Acne	5 (6.0)	11 (11)
	Eczema	6 (7.2)	14 (14)
	Psoriasis	10 (12.0)	13 (12)
	Urticaria	1 (1.2)	1 (1)
	Rosacea	1 (1.2)	1 (1)
	Hidradenitis Suppurativa	10 (12.0)	10 (10)
	Rheumatoid Arthritis	10 (12.0)	10 (10)
	Seronegative Arthritis	1 (1.2)	1 (1)
	Psoriatic Arthritis	2 (2.4)	2 (2)
	Ankylosing Spondylitis	1 (1.2)	1 (1)
	Enteropathic Arthritis	1 (1.2)	1 (1)
	Myeloma	5 (6.0)	5 (5)
	Type 1 Diabetes	10 (12.0)	10 (10)
	Type 2 Diabetes	19 (22.9)	19 (19)
	Ulcerative Colitis	1 (1.2)	1 (1)
EQ-5D-3 L	Baseline	0.738 (0.22)	NA
	Follow-up	0.797 (0.18)	NA
	Change score	−0.059** (0.14)	NA
EQ-VAS	Baseline	59.55 (22.81)	NA
	Follow-up	68.75 (19.83)	NA
	Change score	−9.19 (19.43)	NA
Disease severity (GS scale)	Baseline	5.24 (2.49)	NA
	Follow-up	4.28 (2.45)	NA
	Change score	0.964 (3.11)	NA
**Family member/partner**			
Age	Mean age	50.75 (15.48)	49.25 (14.69)
	Range	18–83	18–83
Gender	Male	37 (44.6)	42 (42)
	Female	46 (55.4)	58 (58)
Ethnicity	White	76 (91.6)	84 (84)
	Asian/Asian British	4 (4.8)	13 (13)
	Black/African/Caribbean/Black British	2 (2.4)	2 (2)
	Prefer not to say	1 (1.2)	1 (1)
Occupation	In paid work	49 (59.0)	64 (64)
	Unemployed	1 (1.2)	1 (1)
	Homemaker	5 (6.0)	5 (5)
	Education/training	1 (1.2)	1 (1)
	Retired	24 (28.9)	24 (24)
	Rather not say	3 (3.6)	5 (5)
Relationship to patient	Spouse/Partner	67 (80.5)	67 (67)
	Parent	6 (7.2)	25 (25)
	Son/Daughter	8 (9.6)	6 (6.1)
	Brother/Sister	1 (1.2)	1 (1.0)
	Other	1 (1.2)	1 (1.0)
FROM-16 scores	Baseline FROM-16	9.54 (6.83)	9.52 (6.57)
	Follow-up FROM-16	8.11 (6.93)	8.55 (7.38)
	Change score	1.43* (5.01)	0.970 (5.41)**
FROM correlation to GRC	Pearson’s correlation	0.39**	0.418 **

** Correlation is significant at < 0.001 level (2-tailed); *Correlation is significant at < 0.05 level (2-tailed); ^a^Paediatric patients, details about occupation not applicable.

*FROM-16* Family Reported Outcome Measure-16 items; *GRC* Global Rating of Change scale; *EQ-5D-3L* Euroqol Five Dimension -three level; *GS* Global severity scale. EQ-5D and EQ-VAS improvement/deterioration runs in the opposite direction to FROM-16. A higher score of FROM-16 means deterioration while a higher score on EQ-5D-3L and EQ-VAS means improvement and vice versa

#### Responsiveness study

The normality of FROM-16 scores at baseline and follow-up was assessed through histograms and Q-Q plots. The skewness (baseline FROM-16 = 0.76; follow-up FROM-16 = 0.86) and kurtosis (baseline = 0.09; follow-up = 0.24) values were within the bounds of normality, indicating normal distribution [26, 27]. Although normality was the basis for choosing the *t*-test versus Wilcoxon, a recent study [37], indicated that when there are more than 25 observations per group and no extreme outliers, the *t*-test works well even for moderately skewed distributions of the outcome variable. Therefore, the study used a *t*-test for analysis. The paired *t*-test was carried out to compare mean FROM-16 family members’ scores and mean patients’ scores for EQ-5D, EQ-VAS, and GSQ between baseline and follow-up. The independent *t*-tests were used to examine gender differences in scores (family member FROM-16 and patient EQ-5D, EQ-Vas, GSQ) at baseline and follow-up.

Although patients and their family members were allowed to choose whether to complete their questionnaires online or by post, all chose to complete the questionnaires online. The overall response rate to the baseline questionnaire was 61.4% (n = 97/158). Of the 97 participants, 86 (88.7%) completed the follow-up, but three patients had not started medication, and hence their responses were not included (Fig. S5). When reminding the respondents, text messaging was the most effective method compared to other methods. Eighty-three patients with 15 different health conditions (mean age = 50.99, SD = 18.71, range = 18-89 years; female 51.8%) and their family members (mean age = 50.75, SD = 15.48; range = 18–83 years; female = 55.4%) were included in the responsiveness analysis (Table 2). Most patients were started on biologics, some were on methotrexate (dermatology and rheumatology), insulin (diabetes) and isotretinoin (dermatology).

There was no statistically significant difference between male and female FROM-16 scores at baseline (females = 10.52, SD = 6.71; male = 8.32, SD = 6.88; ES_Females_ = 0.327, ES_Males_ = 0.319, *p* = 0.146) or at follow-up (females: mean = 8.80, SD = 6.37; males: mean = 7.24, SD = 7.57; ES_Females_ = 0.245, ES_Males_ = 0.206, *p* = 0.311 (Table S1). The mean EQ-5D score for patients at baseline was 0.738 (SD = 0.23), and at follow-up was 0.797 (SD = 0.19) with a mean difference of −0.059 (SD = 0.14, *p* < 0.001) (Table 2). There was no statistically significant difference between patient EQ-5D scores between males and females at baseline (male = 0.75, SD = 0.22; female = 0.73, SD = 0.23; *p* = 0.607) and follow-up (male = 0.82, SD = 17; female = 0.78, SD = 0.20; *p* = 0.376). (Table S1). No floor or ceiling effects were observed for baseline or follow-up FROM-16 scores.

#### MIC study

The overall response rate to the baseline questionnaire was 63% (n = 121) for the MIC study. The follow-up questionnaire was posted to the 121 participants who responded at baseline. In total, 105 (87%) responses were received, with five (4.8%) not eligible because the patient did not start on new medication or change medication, leaving 100 (83%) eligible responses to form the basis of the MIC analysis (Fig. S6).

The family members (mean age = 49.25 years, SD = 14.69; range = 18-83, female = 58%) of patients (mean age = 44.12 years, SD = 22.94, range = 1–89 years, female = 52% with 15 different health conditions were included in the analysis. Two-thirds of the family members were spouses/partners (67%), and a quarter (25%) were parents with 84% from a White background. Family members were mostly in paid jobs (64%), and 24% were retired (Table 2).

### Responsiveness to change over time

The responsiveness analysis, using the paired samples *t* test, showed that the FROM-16 was responsive to change. The mean FROM-16 score of 83 patients at baseline was 9.54 (SD = 6.83) and at follow-up 8.11 (SD = 6.92) with a mean change of 1.43 (*p* < 0.05, *t*-value = 2.6; df = 82) (Tables 2 and 3).

**Table 3:** Testing results of the hypotheses for evaluation of responsiveness of FROM-16 to change over time

**Hypotheses to be tested**	**Family member/partner**				**Patient**			**Hypotheses Confirmed**
	Mean FROM-16 change score (SD)	CC	ES	SRM	Mean EQ-5D^1^ change score (SD)	ES	SRM	
1. An improvement/deterioration in family member/partner QoL (measured by FROM-16) was hypothesised in relation to changes in:								
(a) Patient’s HRQoL on EQ-5D-3 L (n = 83)	1. 43 (5.01)*		0.21	0.29	−0.059 (0.14)**	−0.26	−0.41	Yes
(b) Patient’s disease severity (Improved) on GSQ (n = 42)	2.53 (4.82)**		0.39	0.56	−0.10 (0.14)**	−0.44	−0.73	Yes
(c) Patient’s disease severity (worsened) on GS scale (n = 27)	0.41(5.27)		0.06	0.08	−0.014 (0.17)	−0.06	−0.086	Yes
2. Moderate to high positive correlation between FROM-16 change score and the GRC score		*r* = 0.39**						Yes (moderate)
3. Low to moderate positive correlation FROM-16 and DS change score		*r* = 0.37**						Yes (moderate)
4. Low to moderate negative correlation FROM-16 and EQ-5D change score		*r* = -0.24**						Yes (Low)
5. Mean change improved^a^ is positive (n = 9)	6.89 (8.22)*		0.83	0.85				Yes
6. Mean change worsened^b^ is negative (n = 8)	− 1.38 (2.26)		−0.17	−0.61				Yes
7. Mean change unchanged (n = 6)	1.03 (4.2)		0.17	0.25				Yes, (6.9 > 1.0 > −1.4)
Mean change improved > unchanged > worsened								
8. FROM-16 change score (Improvement)^c^, AUC ≥ 7								Yes, AUC = 0.76*
9. FROM-16 change score (deterioration)^d^, AUC ≥ 7								Yes, AUC = 0.78*

^1^EQ-5D improvement/deterioration runs in the opposite direction to FROM-16; *GRC* Gobal rating of change; *GS scale* Global severity scale; *AUC* area under the receiver operating characteristics curve; *CC* Correlation Coefficient; *ES* Effect size; *SRM* Standardized response mean; *DS* Disease severity; Correlation was interpreted as high (>0.5), moderate (0.3–0.5) or low (<0.3)

** Correlation is significant at < 0.001 level (2-tailed); * Correlation is significant at < 0.05 level (2-tailed)

^a^The mean change score of the family members indicating improvement on the associated GRC scale; ^b^The mean change score of the family members indicating worsening on the associated GRC scale; ^c^based on the anchor perceived improvement; ^d^based on the anchor perceived deterioration

#### Distribution method

The ES of the FROM-16 change score was 0.2 while the SRM was 0.3, both indicating a small effect according to Cohen’s criteria (Table 3).

#### Anchor-based method

There was significant moderate correlation (*r* = 0.39) between the GRC scale and the FROM-16 change scores confirming hypothesis 2 (Table 1). Family members who recorded an improvement (n = 9) on the GRC scale had a positive mean change score of 6.9 (ES = 0.83), and family members who recorded a worsening (n = 8) on the GRC scale had a negative mean score change of  −1.4 (ES = 0.17) (Table 3).

The mean score changes of family members who selected the “no change” option on the GRC had a positive mean FROM-16 change score of 1.07 (ES = 1.9). These results show that ‘mean change improvement’ > ‘Mean Change unchanged’ > ‘worsened’, thus confirming hypotheses 5–7 for responsiveness (Tables 1 and 3).

**Fig. 1:**
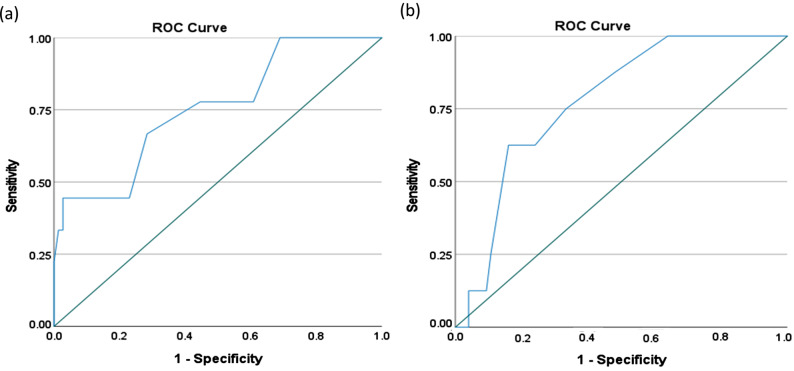
ROC curve indicating responsiveness of FROM-16 (**a**) improvement versus no improvement (**b**) deterioration versus no deterioration

Figure 1 presents the ROC curves generated for the FROM-16 change score based on the anchor perceived improvement and deterioration. The AUC of the FROM-16 was 0.76 (95% CI: 0.58, 0.93; *p* = 0.013) for Improvement and 0.78 (95% CI: 0.64, 0.91; *p* = 0.011) for deterioration confirming hypotheses 8 and 9 (Tables 1 and 3). The AUC was above 0.7 for both improvement and worsening of QoL in family members, indicating good responsiveness.

### Responsiveness of FROM-16 to changes in patient HRQoL

The mean EQ-5D score for patients at baseline was 0.74 (SD = 0.22), and at follow-up was 0.81 (SD = 0.18) with a mean difference of −0.059 (SD = 0.143, *p* < 0.001). The family members’ QoL changed in parallel to the patient’s QoL over three months (Table 3) confirming hypothesis 1 (Table 1). The magnitude of change in patient’s QoL observed through change in EQ-5D scores (ES = 0.263, SRM = 0.412,) was closely related to changes in family member FROM-16 scores (ES = 0.210, SRM = 0.286) indicating a small change in effect size according to Cohen’s criteria (Table 3).

**Fig. 2:**
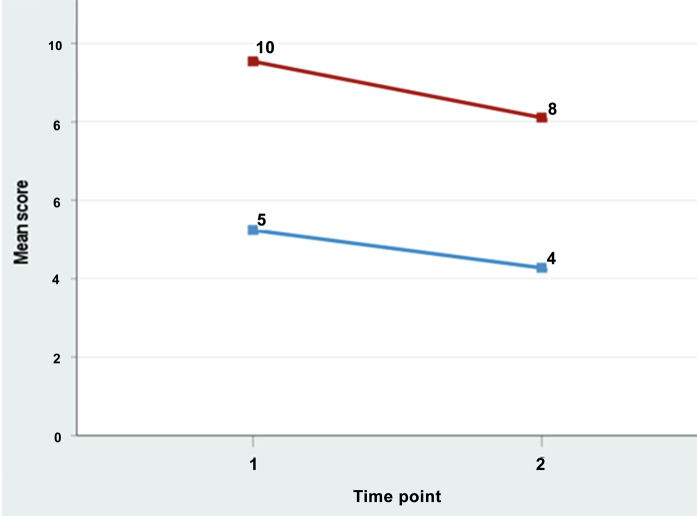
Sensitivity of FROM-16 to patients’ disease severity scores between baseline and follow-up

The mean disease severity (GS Scale) score at baseline was 5.24 (SD = 2.5) and at follow-up 4.28 (SD = 2.4), with a mean change of 0.96 (*p* < 0.05). There was moderate correlation (*r* = 0.37, *p* < 0.05, ES = 0.39) between change score for patient disease severity and FROM-16 change score confirming hypothesis 3 (Table 1). Table 3 shows that as the disease severity improved, QoL was improved in patients with simultaneous improvement in the QoL of family members. However, worsening of disease severity was associated with a small improvement rather than deterioration in QoL both in patients and in family members. Furthermore, there was a positive direct relationship between the patients’ self-assessed disease severity and QoL of family members (Fig. 2). There was a low negative correlation between patient EQ-5D change scores and family members FROM-16 change scores confirming hypothesis 4 (Table 1). Thus, all nine predefined hypotheses (Table 1) concerning FROM-16 responsiveness were met, indicating that FROM-16 can not only measure change in family members’ QoL over time but is also responsive to changes in patients’ HRQoL and disease severity (Table 3).

### Estimation of FROM-16 MIC value

#### ROC curve analysis

**Fig. 3:**
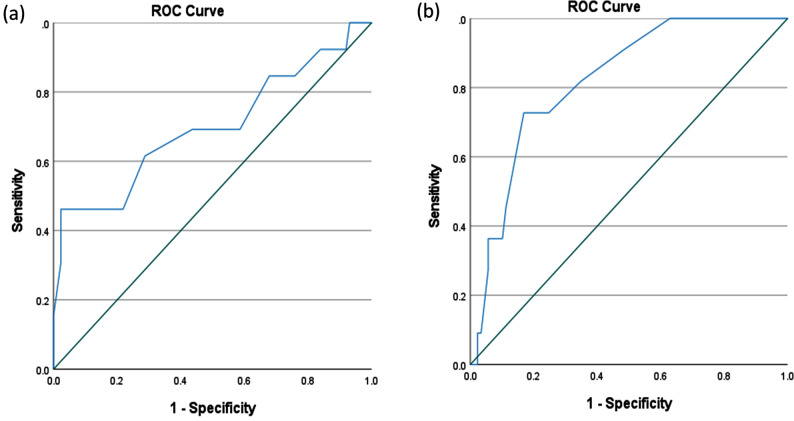
Receiver Operating Curve characteristic curve showing MIC value for (**a**) improvement and (**b**) deterioration for FROM-16

There was significant moderate correlation between FROM-16 change score and GRC scale (*r* = 0.418). The MIC_improvement_ for FROM-16 using ROC curve analysis was estimated as 6.5 (AUC = 0.698, *p* = 0.022, CI = 0.516, 0.880) and MIC_deterioration_ was 1.5 for (AUC = 0.821, *p* = 0.01, CI = 0.710, 0.933). The AUC and its 95% confidence interval is the probability to correctly identify a “meaningful change”. For MIC improvement, the sensitivity was 47.2% and specificity was 97.7% and for MIC deterioration, the sensitivity was 72.7% and specificity was 83.1% (Tables S2 and S3) (Fig. 3).

#### Predictive modelling

The MIC value (MIC_PRED_) was calculated using the logistic regression modelling approach proposed by Terluin et al. [27]. The results of the regression analysis for improvement and deterioration (Table 4a and 4b) and subsequent calculation of adjusted MIC_pred_ using Terluin et al’s formula [34] are shown below:

**Table 4a:** Results of logistic regression analysis for Improvement

	B	SE.	Sig.	Exp(B)	95% CI for EXP(B)	
					Lower	Upper
FROM-16_score change Constant	0.209	0.082	0.011	1.233	1.049	1.449
	−2.423	0.418	<0.001	0.089		

*B* regression coefficient; *SE* standard error; *Sig* Significance <0.05; *Exp(B)* Exponential value of B

**Table 4b:** Results of logistic regression for deterioration

	B	SE.	Sig.	Exp(B)	95% CI for EXP(B)	
					Lower	Upper
FROM-16 score change Constant	0.177	0.061	0.004	0.838	0.744	0.944
	−2.229	0.365	<.001	0.108		

*B* regression coefficient; *SE* standard error; *Sig* Significance <0.05; *Exp(B)* Exponential value of B

##### Calculation of MIC_predict_ improvement

The omnibus (combined test) was significant (*p* = 0.001), indicating that the current model outperforms the null model. Nagelkerke’s R² is 0.2.

MIC_pred_ (improvement) was calculated by substituting values for constant and regression coefficient for FROM-16 score changes given in Table 4a in the formula below:

$$MI{C_{predict}} = \left( {\log \left( {Odd{s_{pre}}} \right) - c} \right)/b$$

ln(0.13/(1–0.13))–2.423/0.209

ln(0.149)–(−2.423)/0.209

(−1.901 + 2.423)/0.209 **= 2.498**

##### Calculation of MIC_predict_ deterioration

The omnibus (combined test) was significant (*p* = 0.002), indicating that the current model outperforms the null model. Nagelkerke’s R² is 0.2

The MIC pred (deterioration) was calculated by substituting values for constant and regression coefficient for FROM-16 score changes given in Table 4b in the formula below:

$$MI{C_{predict}} = \left( {\log \left( {Odd{s_{pre}}} \right) - c} \right)/b$$

ln(0.11/(1–0.11)) − (−2.229)/0.177

ln(0.1236) + 2.229/0.177

(−2.0907 + 2.229)/0.177 = 0.1383/0.1770.781355 = 0.78

##### Calculation for the adjusted MIC_pred_ (improvement)

$$MI{C_{predict(adjusted)}} = MI{C_{predict}} - \left( {0.090 + 0.103*Cor} \right)*S{D_{change}}*\log {\left( {odd{s_{pre}}} \right)_{Improvement}}.$$

MIC_pred(imp)_ = 2.498; Cor = 0.418; SD_change_ = 5.413; log-odds(pred)imp = − 1.901

Therefore, *MIC*_*pred (adjusted)*_ = 2.498 –(0.090 + 0.103*0.418)*5.413*−1.901

=2.498–0.1331 *5.413* −1.901

*=2.498 − (−1.369) = 3.867 = 3.9*

##### Calculation for adjusted MIC_pred_ (deterioration)

$$MI{C_{predict(adjusted)}} = MI{C_{predict}} - \left( {0.090 + 0.103*Cor} \right)*S{D_{change}}*\log {\left( {odd{s_{pre}}} \right)_{Deterioration}}.$$

Here, MICpred(det) = 0.781; Cor = 0.418; SD_change_ = 5.413; log-odds(pred)_det_ = −2.09074 Therefore, MICpred (adjusted)=

0.781 − (0.090 + 0.103*0.418)*5.413* −2.09074

0.781 − (0.1331)*5.413* −2.091 = 2.2867 = **2.3**

The adjusted MIC_pred_ for improvement was 3.9, and for deterioration was 2.3

##### Distribution-based methods

The MIC for FROM-16 applying 0.33*SD gave a value of 2.2, 1 SEM gave a value of 2.2 (1 SEM is equivalent to 0.33 ES when the reliability is 0.9 [38]) and 1.96 SEM gave a value of 4.2 (Table 5).

##### Proposed MIC for use in clinical practice and research scenarios

Based on the results summarised in Table 5, the overall MIC for FROM-16 could lie between 3.1 to 4.2. We have excluded I SEM and taken 1.96 SEM into consideration for calculating MIC as it is a more stringent estimation, representing 95% confidence that this figure is above the measurement error. We excluded 0.33 SD from the triangulation of the results since 0.33 SD is equal in value to 1 SEM when the reliability is 0.9 [38]. Therefore, the final MIC is based on 1.96 SEM, the mean of adjusted MIC for improvement and deterioration based on predictive modelling and the mean of MIC for improvement and deterioration based on ROC analysis. These calculations resulted in the MIC of FROM-16 as 3.76. However, since the FROM-16 score is a whole number, an MIC value of four is suggested for FROM-16.

**Table 5:** Triangulating MIC values from anchor-based and distribution methods

Methods		MIC		
		improvement	deterioration	Overall
Anchor-based	ROC curve analysis (95%CI)^a^	6.5 (1, 8)	1.5 (−2, 4)	**4.0**
	Predictive modelling LR MIC_pred_ (95%CI)^b^	2.5 (−3.6, 6.2)	0.78 (−5.7, 5)	
	Predictive modelling LR^b^ MIC_pred (Adjusted)_ (95%CI)^c^	3.9 (−0.1, 9.5)	2.3 (−2.7, 7.6)	**3.1**
Distribution-based	0.33 SD			2.17 = 2.2
	1 SEM^1^ = SD_b_ *$\sqrt {1 - {\text{Reliability}}}$			2.16 = 2.2
	1.96 SEM			**4.23 = 4.2**
Mean MIC				3.76 = 3.8

“bold values” are included in the MIC analysis

^1^ Cronbach’s Alpha for FROM-16 score was 0.89; *MIC* Minimal Important Change; *SD*_b_ Standard Deviation of the FROM-16 score at the baseline; *SEM* Standard Error of Measurement

^a^based on 1000 bootstrap simulation; ^b^Adjusted for the proportions improved and deteriorated; ^c^CI based on Turluin et al.’s [34] Excel sheet (Figs. S1–S4)

## Discussion

This study confirms for the first time the responsiveness to change over time of FROM-16. While the anchor-based methods involved family members’ perspectives of change in their QoL, the distribution-based method was based on the statistical distribution of QoL scores, providing insight into the magnitude of change that occurred between the assessments. The study results, using the distribution-based approach, indicate that there had been a small change in family members/partners’ QoL over three months following patient treatment with a new medication. This is not surprising given that patients had also experienced a small change in their QoL but within the range of MIC value for EQ-5D-3L [39]. The patients involved in this study were from five different specialities and had 15 different health conditions. Presumably, the treatments and therapies they received were different, and hence, one could expect varying efficacy experienced by the patients and variability in score changes. For example, diabetes patients in this study included not only those with poor glycaemic control starting on insulin treatment but also those who had insulin intensification. Although insulin treatment can have a major effect in controlling patients’ glycaemic levels, it may only have a subtle effect on the QoL of patients and family members because most of them have been living with diabetes for a long time. In contrast, myeloma patients starting on biologics or having transfusions may take longer to see a beneficial qualitative change as many often experience treatment side effects when starting therapy. While this variability in the patients’ responses to treatment may have resulted in an overall small change, it is important to include the full spectrum of a disease severity, from mild to severe, when testing generic tools. Although using the distribution-based method, there was only a small effect size for FROM-16 change over time, FROM-16 responsiveness should be viewed in the context of magnitude of change in the patients’ QoL.

A 15-point GRC scale was used as an external measure to test FROM-16 responsiveness. The GRC scale showed moderate correlation to changes in FROM-16 score (*r* = 0.39, *p* < 0.001). The strength of correlation between anchor and FROM-16 is comparable to other studies on responsiveness (DLQI, *r* = 0.32; numerical pain rating scale, *r* = 0.49; Euroqol, *r* = 0.42) [18, 40, 41], using the same approach. The hypothesis was confirmed that the mean change in FROM-16 scores for the anchor categories (i.e. improvement, deterioration and no change) were ordered in the expected direction. This fulfils one of the criteria for establishing responsiveness of FROM-16. The mean change in FROM-16 scores of those who recorded improvement on the GRC scale was positive, change in scores for family members who recorded worsening was negative, and the mean change in improvement was greater than the mean change in unchanged, which was, in turn, greater than those whose QoL was recorded as worsened on the GRC scale. The effect size for “improvement” was large, indicating excellent responsiveness of FROM-16 to improvement in QoL following a patient starting new treatment. The ROC analysis also demonstrated that FROM-16 was responsive to improvement (AUC = 0.76) and deterioration (AUC = 0.78) in family members’ QoL, as recorded on the GRC scale. Surprisingly, only 17 family members out of 83 recorded any change on GRC scale. The advantage of the 15-point GRC scale is that it granulates the change, helping respondents to select the smallest change experienced. However, most family members recorded ‘no change’ on the GRC scale. This could be attributed to the formatting of the online anchor question, which required participants to answer this question in a two-step process: first, to choose from one of ‘improved’, ‘the same’ or ‘deteriorated’ and then, if improvement or deterioration was chosen, the further detailed options were shown. This design was intended to make the questionnaire simple, but this two-step process initially obscured the multiple options. There is a possibility that presenting the item in two stages might have attenuated possible ratings for the smallest change options. However, results from the anchor-based method are consistent with the responsiveness demonstrated by the distribution method.

Although only a small number of family members fell in the change category, data were analysed for responsiveness using ROC analysis for transparency and for comparison to the results from the distribution-based method. The results from ROC analysis do provide supportive evidence of responsiveness demonstrated by the distribution method. Furthermore, this study used an additional anchor GSQ, measuring patient disease severity, completed by each patient at two assessment points. FROM-16 was responsive to changes in patients’ disease severity between these two assessments. This meant that for the patients who reported improvement (n = 42, ES = 0.44) in disease severity between the assessments, their family members also reported corresponding improvement (n = 42, ES = 0.39). Since FROM-16 measures impact of patient disease on family members, GSQ anchor provides more relevant information about its responsiveness, further confirming the longitudinal validity of FROM-16 in the construct being measured. The parallel improvement in the FROM-16 scores and patients’ GSQ scores, suggests family members’ improvement was directly linked to patients’ improvement and is indicative of how new treatments can improve family members’ QoL. Surprisingly, neither patients’ nor family members’ QoL worsened in response to worsening in disease severity (n = 27) recorded on the GS scale. Instead, a very small improvement was noticed by both the patient and the family member. This suggests that worsening in disease severity, as recorded on the GS scale, might involve a construct not covered by EQ-5D or possibly improvement with a new treatment did not meet patients’ expectations. Perhaps family members had developed coping skills over time or their QoL impact levels were already at the threshold of maximum impact.

The treatment period was chosen as three months as this was thought to be adequate by clinicians to see some change in QoL of patients following treatment. Most patients were started on biologics, some were on methotrexate (dermatology and rheumatology), insulin (diabetes) and isotretinoin (dermatology). Types of biologics used varied across the disease areas. Across five specialities, the HRQoL was the lowest for rheumatology patients and family members of myeloma patients (Table S4). Although rheumatology patients reported moderate improvement, their family members only reported a small improvement in QoL as measured by FROM-16. Only one inflammatory bowel disease (IBD) patient participated in the study, however both the patient and the family member reported an improvement in their QoL (Table S4).

Even though three months is often a standard period for evaluating treatment effect, a longer period might be necessary to notice change in some aspects of QoL in certain conditions. In this study, many patients commenced biologics, with an expected effect within 3–4 months[42], but in other situations it may take much longer to see treatment effects. For example, a study that compared the responsiveness of various care-related QoL measures found that none exhibited clear responsiveness within a year [15].

Our study estimated MIC for FROM-16 for the first time. The sample size for this was bigger (n = 100) than for the responsiveness study (n = 83) because it included additionally data from 17 family members of paediatric patients. The study used both anchor and distribution methods. Anchor-based approaches are generally considered superior as they relate change in scores to an external criterion of important change, thus providing a clinically meaningful estimate of change. Distribution-based methods however provide statistical grounding to the MIC value [13, 43].

The correlation between the GRC outcome and the FROM-16 change score was moderate (*r* = 0.418, *p* = 0.001) and in agreement with guidelines (*r* ≥ 0.3.) when using an anchor-based approach [43]. The ROC method in this study resulted in the MIC value of 6.5 (*p* = 0.02, AUC = 0.698 for improvement and 1.5 (*p* = 0.001, AUC = 0.821) for deterioration. The ROC curves not only compare a continuous scale to a benchmark but also determine if this relationship differs from chance alone, thus combining an anchor-based approach with a distribution-based approach [44]. However predictive modelling method is considered more precise [13]. The MIC_pred_ for the FROM-16 was estimated to be 2.5 for improvement and 0.78 for deterioration. Nevertheless, both methods may be subject to bias if the proportion of improved and not improved is greater or smaller than 50% [13] and in this study the proportion of improved was smaller than 50% which means that the results could have been underestimated. Therefore, this study also calculated adjusted MIC_pred_. The adjusted predictive modelling method (MIC_pred (adjusted)_), allows corrections to this bias [13, 34]. The adjusted MIC_pred,_ using the Terluin formula [34], was 3.9 for improvement and 2.3 for deterioration.

The study also used the distribution method, the 0.33 SD for calculation of MIC value. However, compared to the SD method, SEM is not sample dependent, hence may result in a more reliable MIC value. Threshold values of ‘1’ SEM and ‘1.96’ SEM are proposed to reflect MIC [45]. In this study, values for 1SEM and 1.96 SEM were estimated as 2. 2 and 4.2. The SEM estimates the error associated with the measure, implying that changes below the SEM could result from a measurement error. A disadvantage of distribution methods is that they do not indicate importance of observed change. However, combining anchor-based and distribution-based methods is recommended [45] to take advantage of an external criterion and a measure of variability.

This study used both anchor- and distribution-based methods to estimate the MIC for the FROM-16 and, based on triangulation of such methods, arrived at a single value for the MIC. This is supported by the recent literature review of methods used in estimating the Minimal Clinically Important Difference (MCID) for Health-Related Quality of Life (HRQoL) instruments conducted by Mouelhi et al. [46], who contend that the MCID can be best estimated using a combination of anchor and distribution measures triangulating toward a single value. However, the MIC/MCID value should not be seen as a deterministic cut-off point to interpret score changes but rather a probabilistic value indicating that an individual has experienced a meaningful change [13].

Furthermore, although MIC values were separately calculated for improvement and for deterioration, our intention was to propose a single MIC value for practical purposes when FROM-16 is routinely used. There is a practical need for a reliable single FROM-16 MIC value, despite uncertainly from methodologies suggesting differing results (Table 5), a MIC value of four is proposed. The suggested MIC value of four is closer to the MIC value for improvement (3.9) than to the MIC value for deterioration (2.3) on anchor-based predictive modelling. It would be of great interest to explore this phenomenon further, which is not unique to FROM-16 [47, 48]. Therefore, a future study, using a large sample, should establish whether there is a need for separate MIC values for improvement and deterioration.

This study has several strengths. This study reports the responsiveness to change and MIC value for FROM-16. This is an important contribution given only a few disease specific family QoL measures have confirmed responsiveness and only one disease specific family QoL measure has established MIC value [4]. Second, the study explored several distributional and anchor-based methods, including the more recent method of predictive modelling. Third, this study has followed COSMIN guidelines [11–13], which is a prerequisite for these types of studies. Fourth, in assessing responsiveness of FROM-16 to change, patients were directly involved in reporting QoL changes following the intervention. Other studies have compared patients’ QoL changes with that of family members, but have used proxy reporting by family members [14, 15]. Such proxy reporting does not always match self-reports [49].

The study results have implications for economic evaluation and health technology assessment. This study establishes the longitudinal validity of FROM-16 and suggests that FROM-16 can be used in health economic evaluation to include family member/partner impact. This study not only demonstrated how HRQoL measured by FROM-16 changes over time, but that the change measured was also directly related to the patients’ self-reported disease severity, in the expected direction [50]. The MIC value for FROM-16 can be used by clinicians and researchers as a bench mark to assess the impact of an intervention on family members of patients and as a secondary endpoint in clinical trials of new medications.

In terms of the study limitations, only a small number of family members (17/83 for responsiveness and 24/100 for MIC assessment) recorded changes on the GRC scale. The number of responses of change versus no change was less than 50%, but this bias was corrected by using adjusted MIC_Pred_, to calculate the MIC value [13]. The design of the online GRC question initially obscured the wider options from the family members, possibly increasing the number of “no change” answers. Perhaps the majority of family members really did not experience change in their QoL, as suggested by the small ES of change noted in both patients and family members. Future studies should be of bigger sample size to increase the change group numbers. Our sample size was modest but within recommended parameters: ≥50 subjects for responsiveness and ≥100 for MIC [12, 31]. Another limitation was the use of GRC as an anchor. Even though GRC scales are considered the best single measure of the importance of change from the patient’s perspective, it may not provide a correct assessment of change as perception of change is dependent on the subjective experiences of a person, which can be impacted by a number of factors beyond disease impact. Furthermore, GRC is subject to recall bias however, a clinical endpoint was not suitable as subjects had a range of health conditions. Nonetheless, GRC scales have been proven to be sensitive to both positive and negative changes [51, 52].

## Conclusions

The results of this study establish the responsiveness and longitudinal validity for the FROM-16. A MIC value of four is proposed for FROM-16, allowing clinicians and researchers to judge the effectiveness of interventions that may influence family member’s QoL.

### Electronic supplementary material

Below is the link to the electronic supplementary material.

Supplementary Material 1

## Data Availability

The data are available from the authors on reasonable request according to Cardiff University regulations.

## Abbreviations

AUC: Area under the curve
COSMIN: COnsensus-based Standards for the selection of health Measurement Instruments
EQ-5D-3L: European quality of life five dimension- 3- level
FROM-16: Family Reported Outcome Measure
GRCQ: Global rating of change question
GSQ: Global severity question
MIC: Minimal important change
ROC: Receiver operating characteristic
SD: Standard deviation
SEM: Standard error of the measurement
SRM: Standardized response mean
