# Supplementary material for: Responsiveness and minimal important change of the Family Reported Outcome Measure (FROM-16)
Source: J Patient Rep Outcomes. 2024 Mar 26;8:38. doi: 10.1186/s41687-024-00703-1 (PMC10965873; doi:10.1186/s41687-024-00703-1)

Supplementary material

Responsiveness and Minimal Important Change of the Family Reported Outcome Measure (FROM-16)

INDEX: Tables/figures included in the online resource

Table S1 Independent t-test for differences between male and female genders
Table S2 Classification Table for MIC improvement
Table S3 Classification Table for MIC deterioration
Table S4Change in scores in patients and their family members across medical specialities
Figure S1-S4 Confidence calculation of MIC_{Pred} and Adj_MIC_{pred}
Figure S5-S6 Flow diagram for participant recruitment

Table S1 Independent t-test for differences between male and female genders

	Mean		Mean difference	p-value *
	Male Mean(SD)	Female Mean(SD)		
**Family members**	(n=37)	(n=46)		
B_FROM	8.32 (6.88)	10.52 (6.71)	-2.20	0.146
F_FROM	7.24 (7.57)	8.80 (6.37)	-1.56	0.311
**Patients**	(n=40)	(n=43)		
B_EQ-5D	0.75 (0.22)	0.73 (0.23)	0.02	0.607
F_EQ-5D	0.82 (0.17)	0.78 (0.20)	0.04	0.376
B_EQ-5D-VAS	60.25 (23.72)	58.91 (22.19)	1.34	0.791
F_EQ-5D-VAS	69.83 (18.64)	67.74 (21.06)	2.09	0.636
B_GSQ	5.23 (2.81)	5.26 (2.17)	-0.03	0.956
F_GSQ	3.95 (2.33)	4.58 (2.54)	-0.63	0.242

*Significant at < 0.05 level.

Table S2. Classification Table MIC improvement

Classification Table MIC improvement					
Cut point	Sensitivity	Specificity	1-Sensitivity	1 - Specificity	(1-sensitivity] + [1-specificity])
-17	1	0	0	1	1
-15.5	1	0.011	0	0.989	0.989
-14.5	1	0.023	0	0.977	0.977
-12.5	1	0.034	0	0.966	0.966
-10.5	1	0.046	0	0.954	0.954
-8	1	0.069	0	0.931	0.931
-5.5	0.923	0.08	0.077	0.92	0.997
-4.5	0.923	0.092	0.077	0.908	0.985
-3.5	0.923	0.138	0.077	0.862	0.939
-2.5	0.923	0.161	0.077	0.839	0.916
-1.5	0.846	0.241	0.154	0.759	0.913
-0.5	0.846	0.322	0.154	0.678	0.832
0.5	0.692	0.414	0.308	0.586	0.894
1.5	0.692	0.563	0.308	0.437	0.745
2.5	0.615	0.713	0.385	0.287	0.672
3.5	0.462	0.782	0.538	0.218	0.756
4.5	0.462	0.851	0.538	0.149	0.687
5.5	0.462	0.931	0.538	0.069	0.607
6.5	0.462	0.977	0.538	0.023	0.561
7.5	0.308	0.977	0.692	0.023	0.715
9	0.231	0.989	0.769	0.011	0.78
10.5	0.154	1	0.846	0	0.846
18.5	0.077	1	0.923	0	0.923
27	0	1	1	0	1

Table S3 Classification Table MIC improvement

Classification Table MIC deterioration					
Cut point	Sensitivity	Specificity	1-Sensitivity	1 - Specificity	(1-sensitivity] + [1-specificity])
-27	1	0	0	1	1
-18.5	1	0.011	0	0.989	0.989
-10.5	1	0.022	0	0.978	0.978
-9	1	0.045	0	0.955	0.955
-7.5	1	0.067	0	0.933	0.933
-6.5	1	0.09	0	0.91	0.91
-5.5	1	0.135	0	0.865	0.865
-4.5	1	0.213	0	0.787	0.787
-3.5	1	0.281	0	0.719	0.719
-2.5	1	0.371	0	0.629	0.629
-1.5	0.909	0.517	0.091	0.483	0.574
-0.5	0.818	0.652	0.182	0.348	0.53
0.5	0.727	0.753	0.273	0.247	0.52
1.5	0.727	0.831	0.273	0.169	0.442
2.5	0.455	0.888	0.545	0.112	0.657
3.5	0.364	0.899	0.636	0.101	0.737
4.5	0.364	0.944	0.636	0.056	0.692
5.5	0.273	0.944	0.727	0.056	0.783
8	0.182	0.955	0.818	0.045	0.863
10.5	0.091	0.966	0.909	0.034	0.943
12.5	0.091	0.978	0.909	0.022	0.931
14.5	0	0.978	1	0.022	1.022
15.5	0	0.989	1	0.011	1.011
17	0	1	1	0	1

Table S4 Change in scores in patients and their family members across medical specialities

IBD, Inflammatory Bowel Disease; ES, Effect Size; SRM, Standard Response Mean

	Family Member/Partner					Patient				
**Medical specialty**	**B_FROM**	**F_FROM**	**Mean diff FROM**	**ES**	**SRM**	**B_EQ-5D**	**F_EQ-5D**	**Mean diff EQ-5D**	**ES**	**SRM**
**Dermatology (n=33)**	9.03	7.12	1.91	0.258	0.310	0.71	0.80	-0.09	-0.374	-0.552
**Diabetes (n=29)**	8.38	6.93	1.45	0.242	0.424	0.79	0.80	-0.01	-0.056	-0.125
**Rheumatology (n=15)**	11.40	11.27	0.13	0.022	0.024	0.68	0.76	-0.08	-0.489	-0.517
**Haematology (n=5)**	13.60	12.60	1.00	0.101	0.365	0.78	0.79	-0.01	-0.099	-0.447
**IBD (n=1)**	12.00	5.00	7.00			0.81	1.00	0.19		

Confidence Interval calculation of MIC_{pred} and Adj_MIC_{pred}

We entered the output of the regression analysis into the Excel worksheet given by Terulin and colleagues in 2015 (Available at: 1-s2.0-S0895435615001602-mmc2.xlsx (live.com)

to obtain confidence interval values for MIC pred and Adj MIC predict. Below are screenshots of Excel sheet for confidence interval calculation for MIC pred and Adj_MIC pred for Improvement and Deterioration.

Figure S1 Calculating 95% confidence intervals MICpred (Improvement)

1				
2	Prevalence (0-1)	0.130		
3	Change score (X) =	2.498		
4	Intercept (C) =	-2.423	se(C) =	0.418
5	Regression coeff (B)=	0.209	se(B) =	0.082
6			r(C-B) =	-0.648
7	ln(odds-post) =	-1.901	se(ln(oddsp	0.325
8	odds(post)* =	0.149		
9	odds(pre) =	0.149		
10	ln(odds-pre)	-1.901		
11	ln(oddspost)lo =	-1.264	odds(post)l	0.283
12	ln(oddspost)hi =	-2.538	odds(post)l	0.079
13				
14	LR =	1.000		
15	LR(upper limit) =	1.891		
16	LR(lower limit) =	0.529		
17				
18	prob-post =	0.130		
19	prob-post(lo) =	0.220		
20	prob-post(hi) =	0.073		
21				
22	X for which LR=1	2.498		
23	X for which LR(upper limit)=1	-3.574		
24	X for which LR(lower limit)=1	6.241		
25				
26				

Template Terluin | **MICpred(Imp)** | ADJ_MICPred(imp) | MICpred(Det) | ADJ_MICpred(Det)

Figure S2 Calculating 95% confidence intervals Adj_MICpred (Improvement)

1				
2				
3	Prevalence (0-1)	0.130		
4	Change score (X) =	3.867		
5	Intercept (C) =	-2.423	se(C) =	0.418
6	Regression coeff (B)=	0.209	se(B) =	0.082
7			r(C-B) =	-0.648
8	ln(odds-post) =	-1.615	se(ln(oddsp	0.322
9	odds(post)* =	0.199		
10	odds(pre) =	0.199		
11	ln(odds-pre)	-1.615		
12	ln(oddspost)lo =	-0.984	odds(post)	0.374
13	ln(oddspost)hi =	-2.245	odds(post)	0.106
14				
15	LR =	1.000		
16	LR(upper limit) =	1.879		
17	LR(lower limit) =	0.532		
18				
19	prob-post =	0.166		
20	prob-post(lo) =	0.272		
21	prob-post(hi) =	0.096		
22				
23	X for which LR=1	**3.867**		
24	X for which LR(upper limit)=1	-0.105		
25	X for which LR(lower limit)=1	9.469		
26				
27				
28				
29				

Template Terluin | MICpred(Imp) | **ADJ_MICPred(imp)** | MICpred(Det) | ADJ_MICpred(Det)

Figure S3 Calculating 95% confidence intervals MICpred (deterioration)

	A	B	C	D	E	F	G	H	I
1									
2		Prevalence (0-1)			0.110				
3		Change score (X) =			0.7811				
4		Intercept (C) =			-2.229		se(C) =	0.365	
5		Regression coeff (B)=			0.177		se(B) =	0.061	
5							r(C-B) =	-0.349	
7		ln(odds-post) =			-2.091		se(ln(oddsp	0.351	
3		odds(post)* =			0.124				
9		odds(pre) =			0.124				
0		ln(odds-pre)			-2.091				
1		ln(oddspost)lo =			-1.402		odds(post)	0.246	
2		ln(oddspost)hi =			-2.779		odds(post)	0.062	
3									
4		LR =			1.000				
5		LR(upper limit) =			1.991				
6		LR(lower limit) =			0.502				
7									
8		prob-post =			0.110				
9		prob-post(lo) =			0.197				
0		prob-post(hi) =			0.058				
1									
2		X for which LR=1			0.781				
3		X for which LR(upper limit)=1			-5.700				
4		X for which LR(lower limit)=1			5.070				
5									
6									
7									
8									
9									

Template Terluin | MICpred(Imp) | ADJ_MICPred(imp) | **MICpred(Det)** | ADJ_MICpred(Det)

Figure S4 Calculating 95% confidence intervals Adj_MICpred (deterioration)

	A	B	C	D	E	F	G	H	I	J
1		Prevalence (0-1)			0.110					
2		Change score (X) =			2.287					
3		Intercept (C) =			-2.229		se(C) =	0.365		
4		Regression coeff (B)=			0.177		se(B) =	0.061		
5							r(C-B) =	-0.349		
6		ln(odds-post) =			-1.824		se(ln(oddspost)) =	0.342		
7		odds(post)* =			0.161					
8		odds(pre) =			0.161					
9		ln(odds-pre)			-1.824					
10		ln(oddspost)lo =			-1.153		odds(post)lo =	0.316		
11		ln(oddspost)hi =			-2.495		odds(post)hi =	0.082		
12										
13		LR =			1.000					
14		LR(upper limit) =			1.956					
15		LR(lower limit) =			0.511					
16										
17		prob-post =			0.139					
18		prob-post(lo) =			0.240					
19		prob-post(hi) =			0.076					
20										
21		X for which LR=1			2.287					
22		X for which LR(upper limit)=1			-2.687					
23		X for which LR(lower limit)=1			7.594					
24										
25										
26										
27										
28										
29										

Template Terluin | MICpred(Imp) | ADJ_MICPred(imp) | MICpred(Det) | **ADJ_MICpred(Det)**

Figure S5: *Flow chart of recruitment: Baseline to follow-up (Responsiveness study)*

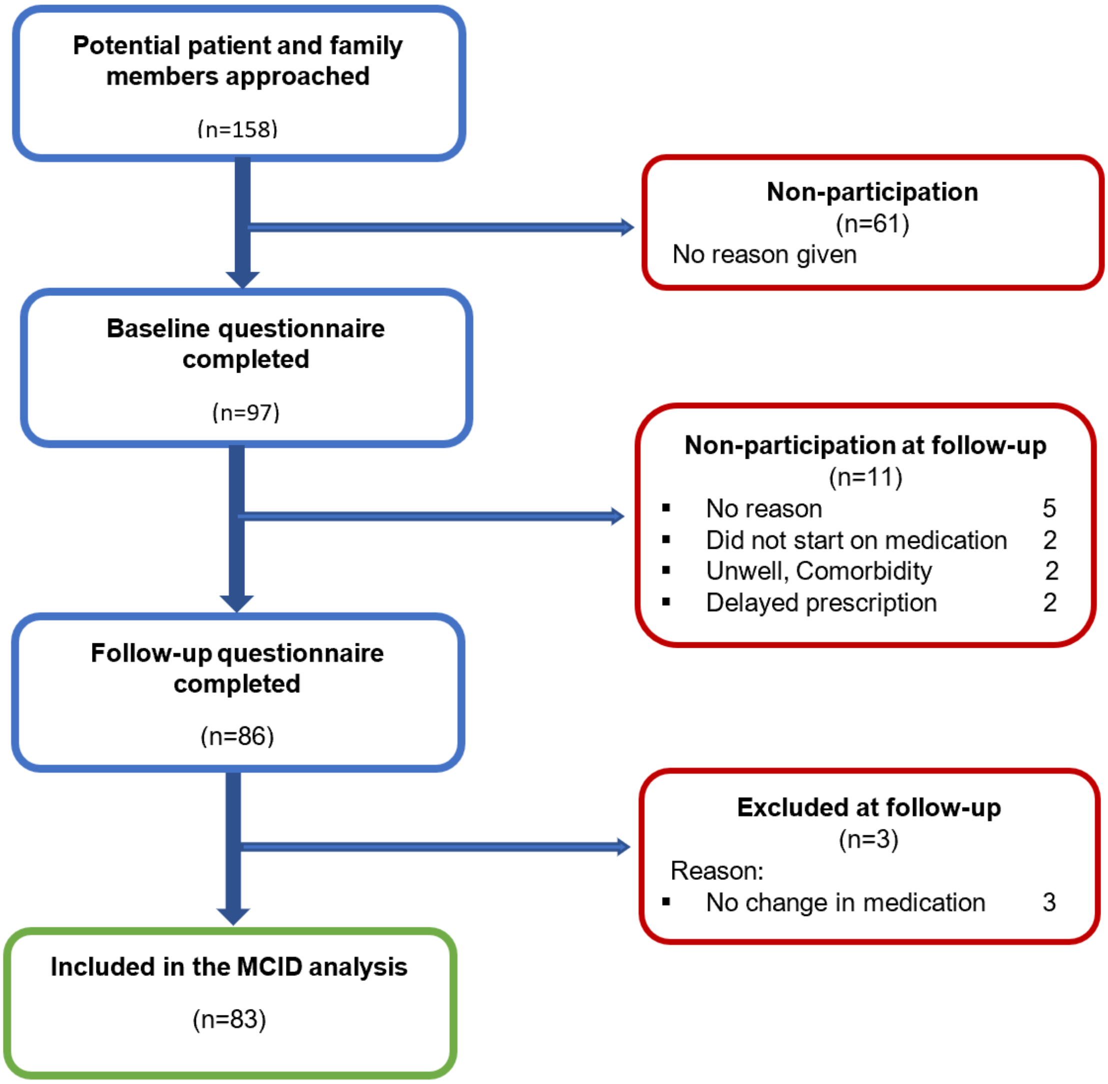

Figure S6: *Flow chart of recruitment: Baseline to follow-up (MIC study)*

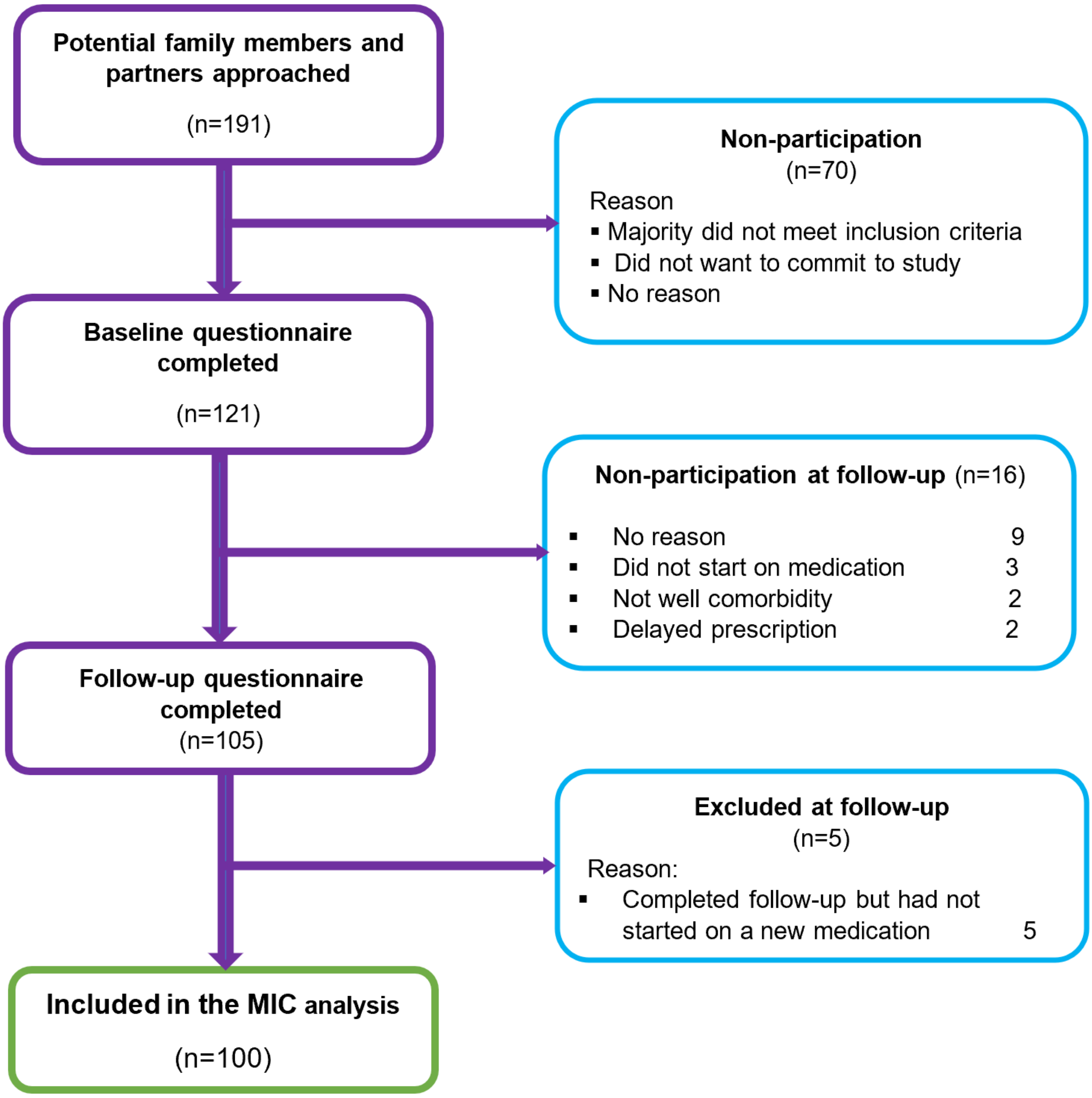

Supplement: Supplementary file 1 — Supplementary Material 1 [file 41687_2024_703_MOESM1_ESM.pdf]
